# Radiologic and clinical changes after denosumab treatment for giant cell tumors of the mobile spine: a quantitative study

**DOI:** 10.1186/s13244-022-01226-3

**Published:** 2022-05-26

**Authors:** Bei Yuan, Songbo Han, Shaomin Yang, Lihua Zhang, Liang Jiang, Feng Wei, Huishu Yuan, Xiaoguang Liu, Zhongjun Liu

**Affiliations:** 1 Department of Orthopaedics, Peking University Third Hospital, No. 49 North Garden Road, Haidian District, Beijing, 100191 China; 2 Department of Orthopaedics, Beijing Tsinghua Changgung Hospital, School of Clinical Medicine, Tsinghua University, Beijing, 102218 China; 3 Department of Radiology, Peking University Third Hospital, Haidian District, No. 49 North Garden Road, Beijing, 100191 China; 4 Department of Pathology, Peking University Third Hospital, No. 49 North Garden Road, Haidian District, Beijing, 100191 China; 5 Engineering Research Center of Bone and Joint Precision Medicine, Beijing, China; 6 Beijing Key Laboratory of Spinal Disease Research, Beijing, China

**Keywords:** Denosumab, Giant cell tumor, Shrinkage, Osteogenesis, Magnetic resonance imaging

## Abstract

**Objectives:**

To analyze the radiologic and clinical changes after denosumab treatment in patients with giant cell tumors (GCTs) in the mobile spine.

**Methods:**

Clinical data and images by computed tomography and magnetic resonance imaging at a single center were retrospectively reviewed before and after denosumab treatment.

**Results:**

Pre- and post-treatment data from 24 patients were evaluated. On imaging, marginal ossification and/or bone formation was observed in 22 patients (91.7%). The median maximum diameter of the GCT reduced from 52.5 to 48.2 mm (*p* < 0.001), and the mean proportion of tumor to spinal canal area decreased from 36.8 to 18.5% (*p* < 0.001). Out of six patients with compression, three patients (50%) showed no compression after treatment. The signal intensity (SI) ratio between the solid part of the tumor and the normal spinal cord on T2-weighted MR images was 0.77 ± 0.22 and decreased to 0.58 ± 0.22 (*p* = 0.001). On clinical symptoms, the mean visual analog scale scores were reduced from 5.3 to 2.0 (*p* < 0.001) and the Karnofsky Performance Scale scores increased from a median of 65 to 80 (*p* < 0.001). Post-treatment, performance scores improved in eight patients (33.3%) (*p* = 0.003), and the neurological function of four patients improved according to Frankel grade (*p* = 0.046).

**Conclusions:**

Bone formation, tumor reduction, regression of epidural lesion and the decrease in SI ratio on T2-weighted image should be considered as the effectiveness of denosumab in the treatment of spinal GCT. In clinical application, denosumab can relieve pain, improve neurological function, and improve the quality of life of spinal GCT patients.

## Key points

- Denosumab is effective for the treatment of GCTs in the mobile spine.
- On images, bone formation, tumor reduction, and SI ratio decrease can be observed.
- Denosumab can relieve pain and improve neurological function of spinal GCT patients.

## Background

Giant cell tumors (GCTs) of the bone are relatively common primary benign bone tumors, accounting for approximately 5% of all primary bone tumors in Western populations [1] and 20% in East Asian populations [2]. In the mobile spine, the reported incidence ranges from 1.4 to 9.4% [3].

Surgery, including curettage, intralesional excision, and en bloc resection, is the primary treatment option in clinical practice [4, 5]. To maintain joint function when GCTs are located in the extremities, extensive curettage with local adjuvants is the first choice for treatment [6]. When GCTs are located in the mobile spine, en bloc resection with wide margins is preferred to minimize local recurrence [7]. However, en bloc resection for spinal GCTs may result in severe morbidity associated with bleeding, infection, and neurological deficits. Moreover, it is not always possible to achieve a tumor-free margin [8].

Denosumab, a fully human monoclonal antibody against the receptor activator of nuclear factor-κB (RANK) and RANK ligand (RANKL), has been approved for patients with GCTs since 2013 [9]. Some phase II clinical trials have demonstrated that up to 86%–88% of patients with GCTs in the extremities respond well to denosumab [10, 11]. Specifically, after the continuous injection of denosumab, patients experienced pain relief, tumor size reduction, and bone formation inside the tumor and/or at its peripheral rim [11–13]. For GCTs of the spine, denosumab is widely used as a preoperative treatment to shrink and ossify the tumor, resulting in less bleeding and the easier manipulation of the consolidated tumor mass during surgery [14]. Denosumab is also used as a stand-alone treatment to inhibit tumor progression and achieve neurological recovery in patients with a high risk for morbidities and/or unresectable lesions [14].

There are currently only a few case reports on radiologic and clinical changes after denosumab treatment for GCTs of the mobile spine. Most of these available reports are limited to descriptions of the phenomenon and lack quantitative analyses [15–18]. This study aimed to add to this knowledge by systematically evaluating the radiologic and clinical changes associated with the use of denosumab in 24 patients with spinal GCTs from a single institution.

## Methods

### Study participants

The study design was approved by the hospital ethics committee, and the need for patient consent was waived owing to the de-identified retrospective review of data. Data were collected from 29 patients diagnosed with GCTs of the spine who received denosumab treatment in our hospital from June 2014 to May 2020. Inclusion criteria included the pathological diagnosis of spinal GCT, complete clinical information, and radiologic images before and after denosumab treatment. Exclusion criteria included pathological diagnosis other than GCT, lack of complete pretreatment clinical information or images, less than 3-month follow-up after initial denosumab injection, or loss to final follow-up. Finally, 24 patients were included in this study (of the remaining patients, three patients lacked complete clinical and imaging data, and two patients were lost to follow-up).

### Clinical information

Data regarding patient demographics, duration of symptoms, neurological status, Enneking and Weinstein–Boriani–Biagini (WBB) grades, treatment history, and treatment regimen were collected. The severity of pain in patients was evaluated using the visual analog scale (VAS), neurological status was evaluated by Frankel grade, quality of life was evaluated using the Karnofsky Performance Scale (KPS), and performance status was evaluated using the Eastern Cooperative Oncology Group (ECOG) scale. All data were collected before denosumab treatment and after the last treatment before surgery.

### Treatment protocol

Patients diagnosed with GCT received subcutaneous denosumab doses of 120 mg monthly (every 28 days), loading doses on days 8 and 15 of the first month, and ingested 400 IU/d vitamin D and 500 mg/d calcium. All 24 patients received at least four doses of denosumab before surgery as preoperative preparation. Of these, six patients received denosumab after surgery for unresectable GCT lesions. Any side effects, including fever, nausea, fatigue, weakness, headache, musculoskeletal pain, back pain, dyspnea, anemia, hypocalcemia, hypophosphatemia, and osteonecrosis of the jaw (ONJ), were recorded. The details of the operation, including the date, procedure type, surgical margin, perioperative and postoperative complications, and postoperative adjuvant treatment, were also collected.

### Radiologic evaluation

Pre- and post-treatment images before surgery were compared and analyzed by an experienced radiologist and orthopedist. The first CT and MRI examinations were performed 1 month after the denosumab injection and every two months thereafter until the last treatment. All 24 patients underwent CT examinations, and 20 patients underwent MRI examinations. All images were independently and blindly evaluated by two readers.

Computed tomography (CT) scans were performed with a GE Light Speed 64-slice CT scanner (General Electric, Boston, MA, USA) with a 120-kV tube voltage, 200–300-mA tube current, 3-mm slice thickness, 3-mm interval, and pitch = 1. Magnetic resonance imaging (MRI) was conducted using the 3-T Magnetom Trio (Siemens, Munich, Germany) and body phased-array coils with patients in the supine position. Slice thicknesses and slice gaps of 3 mm and 0.8 mm, respectively, were utilized for all procedures.

The presence of a peripheral bony rim and bone formation inside the mass, the maximum diameter of the tumor, the height of the vertebral body, proportion of the area of the spinal canal occupied by the tumor, diameter of lung metastasis, size of the cystic component, the signal intensity (SI) ratio between the solid part of the GCTB and the normal spinal cord, and the presence of spinal cord compression were evaluated before and after denosumab treatment.

The degree of tumor ossification was classified into six grades proposed by Boriani et al. [14] To determine the presence of a peripheral rim or bone formation inside the mass, the axial CT scans were evaluated. The peripheral rim results were described as no peripheral rim, incomplete peripheral rim, and complete peripheral rim. Bone formation inside the mass was described as no bone formation, less than 50%, and more than 50% of the transverse session ossified. The resulting classifications were: (1) level 0 with no peripheral rim and no bone formation; (2) level 1a with incomplete peripheral rim and no bone formation; (3) level 1b with complete peripheral rim and no bone formation; (4) level 2a with bone formation inside the mass and less than 50% of the transverse session (any peripheral rim); (5) level 2b with bone formation inside the mass and more than 50% of the transverse session ossified (any peripheral rim); and (6) level 3 with more than 50% of the transverse session ossified and complete peripheral bone formation.

**Fig. 1:**
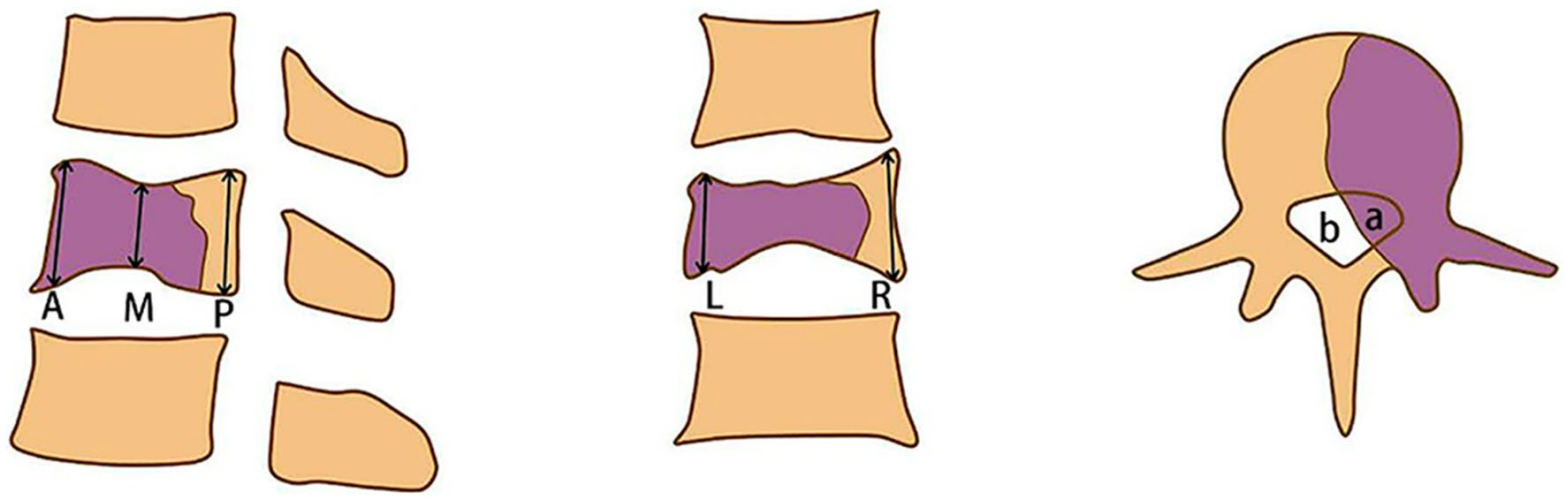
Illustration of the measuring method. Height of the vertebral body was measured on the median sagittal and median coronal CT scans by measuring the height of the anterior (A), posterior (P), middle (M), left (L), and right (R) edge of the vertebral body and take the average. The proportion of the area of the spinal canal occupied by the tumor (*a*/[*a* + *b*]) was evaluated on axial MR images

The maximum diameter of the tumor and lung metastasis was measured on the axial CT scan, and the height of the vertebral body was measured on the sagittal CT scans. The proportion of the spinal canal occupied by the tumor (Fig. 1) and the size of the cystic component were evaluated on axial MR images. SI ratio between the solid part of the GCTB and the normal spinal cord was measured on T2-weighted MR images.

### Statistics

All collected data were analyzed using SPSS 22.0 (IBM Corp; Armonk, NY, USA). Categorical variables are presented as frequencies and percentages, and continuous variables were presented as mean ± standard deviations. Continuous variables were compared using the paired-samples t test or paired-samples Wilcoxon signed-rank test, and categorical variables were compared using the paired-samples Wilcoxon signed-rank test. Significance was set a priori at *p* < 0.05 for all analyses.

## Results

### Demographic data

Twenty-four patients met the study criteria, including eight men and 16 women. All patients had a definitive pathological diagnosis based on CT-guided percutaneous biopsies and/or open surgical procedures. The mean age at diagnosis was 35.3 years (range 13–71 years). All 24 patients had a single spinal lesion, including one patient with lung metastases. In total, 3, 14, and 7 lesions originated in the cervical, thoracic, and lumbar spine, respectively. All lesions were classified as Enneking stage 3.

The mean time between symptom presentation and clinical diagnosis was 6.1 months (range 0.6–24 months). Local pain was the most common symptom (21/24, 87.5%). Fifteen patients (62.5%) had neurological symptoms including radiculopathy (12 patients) and myelopathy (6 patients).

### Treatment

Of the 24 patients enrolled in the study, there were 21 patients with primary tumors and three patients with recurrent tumors who were referred to the study site. After an average of 7.3 doses of denosumab treatment (range 4–21 doses), 12 patients underwent piecemeal intralesional total spondylectomy, six patients underwent total en bloc spondylectomy, and six patients underwent posterior decompression and stabilization or only stabilization for unresectable lesions followed by long-term denosumab injections.

### Radiologic changes

**Fig. 2:**
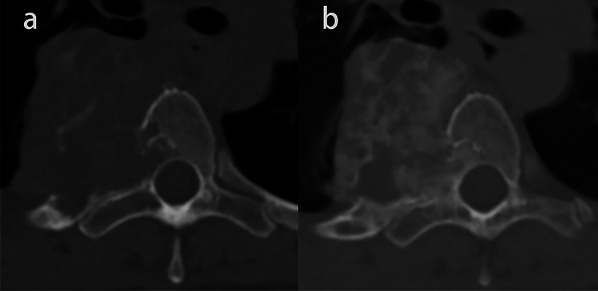
Giant cell tumor of T4 in a 40-year-old woman. **a** Axial CT scan before denosumab treatment. **b** Axial CT scan after denosumab treatment shows a complete peripheral rim and more than 50% of the transverse session ossified (grade 3)

After denosumab treatment, 22 of 24 patients (91.7%) developed marginal ossification, of which 14 patients (58.3%) showed a complete peripheral rim on axial CT scan. Bone formation inside the mass was observed in 19 of 24 patients (79.2%), and 12 patients (50%) showed more than 50% of the transverse session ossified (Fig. 2). Only two of 24 patients (8.3%) did not show any significant ossification. According to the Boriani grading system, two cases were grade 0, one case was grade 1a, two cases were grade 1b, seven cases were grade 2a, three cases were grade 2b, and nine cases were grade 3.

**Fig. 3:**
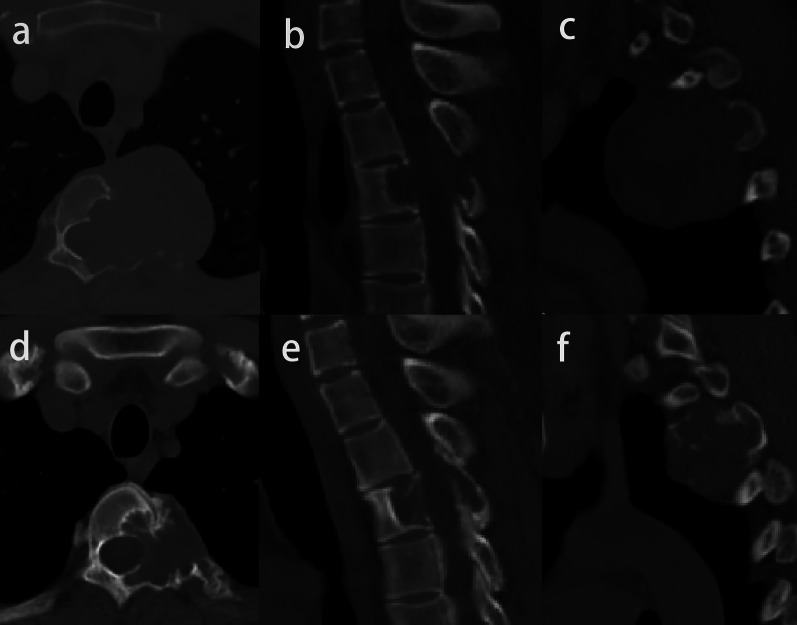
Image of giant cell tumor of T3 in a 30-year-old man before (**a**, **b**, **c**) and after denosumab treatment (**d**, **e**, **f**). The maximum diameter of the tumor reduces from 52.2 mm to 38.0 mm, and the vertebral height decreases from 17.0 mm to 15.9 mm

The median maximum diameter of the tumor was reduced from 52.5 mm (mean, 58.3 mm; range 30.5–147.0 mm) to 48.2 mm (mean, 52.1 mm; range 30.3–90.1 mm) (*p* < 0.001). A total of 21 patients (87.5%) had a reduced tumor size (Fig. 3), and the other three patients had the same tumor size before treatment.

**Fig. 4:**
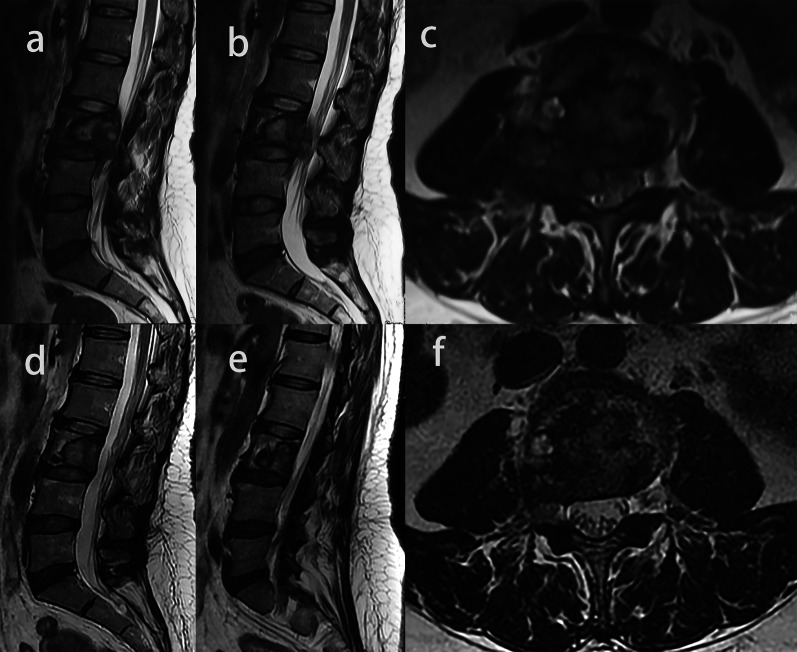
Image of a giant cell tumor of L3 in a 48-year-old woman. **a**, **b**, **c** Axial and sagittal T2-weighted MR images showed epidural lesion before denosumab treatment. **d**, **e**, **f** Axial and sagittal T2-weighted MR images after denosumab treatment. Epidural lesion disappeared completely, and the proportions of the area of the spinal canal occupied by the tumors decreased

**Fig. 5:**
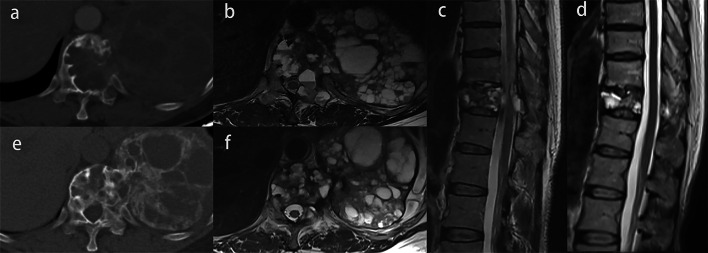
Image of a giant cell tumor of T11 in a 50-year-old woman. **a**, **e** Axial CT scans before and after denosumab treatment. Complete peripheral rim and bone formation inside the mass over 50% of the transverse session are observed (grade 3). **b**, **f** Axial MR images before and after denosumab treatment. The maximum diameter of the tumor and the proportion of the area of the spinal canal occupied by the tumors are smaller. **c**, **d** Sagittal MR images before and after denosumab treatment. The spinal cord compression completely disappears

Twenty patients showed lesions extending to the spinal canal, and the mean proportion of the area of the spinal canal occupied by the tumor decreased from 36.8 to 18.5% (*p* < 0.001) (Figs. 4 and 5). Spinal cord compressions were observed in six out of 24 patients before treatment, and of these six, three patients (50%) showed the epidural lesions shrank or disappeared with no compression after denosumab therapy (Fig. 5), the remaining three patients still had spinal cord compression despite the reduction in the epidural lesions.

**Fig. 6:**
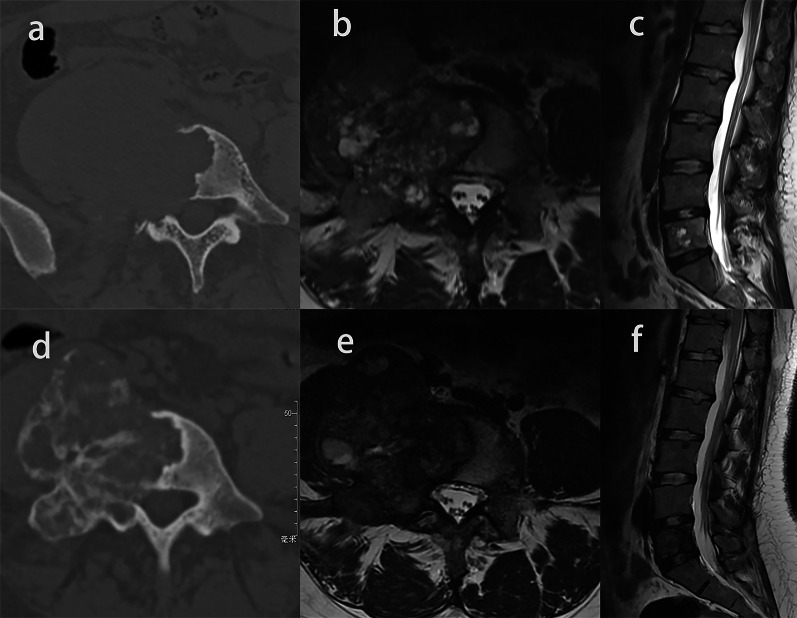
Image of a giant cell tumor of L5 in a 23-year-old woman. **a**, **d** Axial CT scans before and after denosumab treatment. Incomplete peripheral rim and bone formation inside the mass over 50% of the transverse session are observed (grade 2b). **b**, **e** Axial and sagittal MR images before and after denosumab treatment. The diameter of the cyst is smaller. **c**, **f** Sagittal MR images before and after denosumab treatment, the signal intensity (SI) ratio between the solid part of the GCTB and the normal spinal cord on T2-weighted MR images was decreased

The signal intensity (SI) ratio between the solid part of the GCTB and the normal spinal cord on T2-weighted MR images was 0.77 ± 0.22 and decreased to 0.58 ± 0.22 after denosumab treatment (*p* = 0.001) (Fig. 6).

**Fig. 7:**
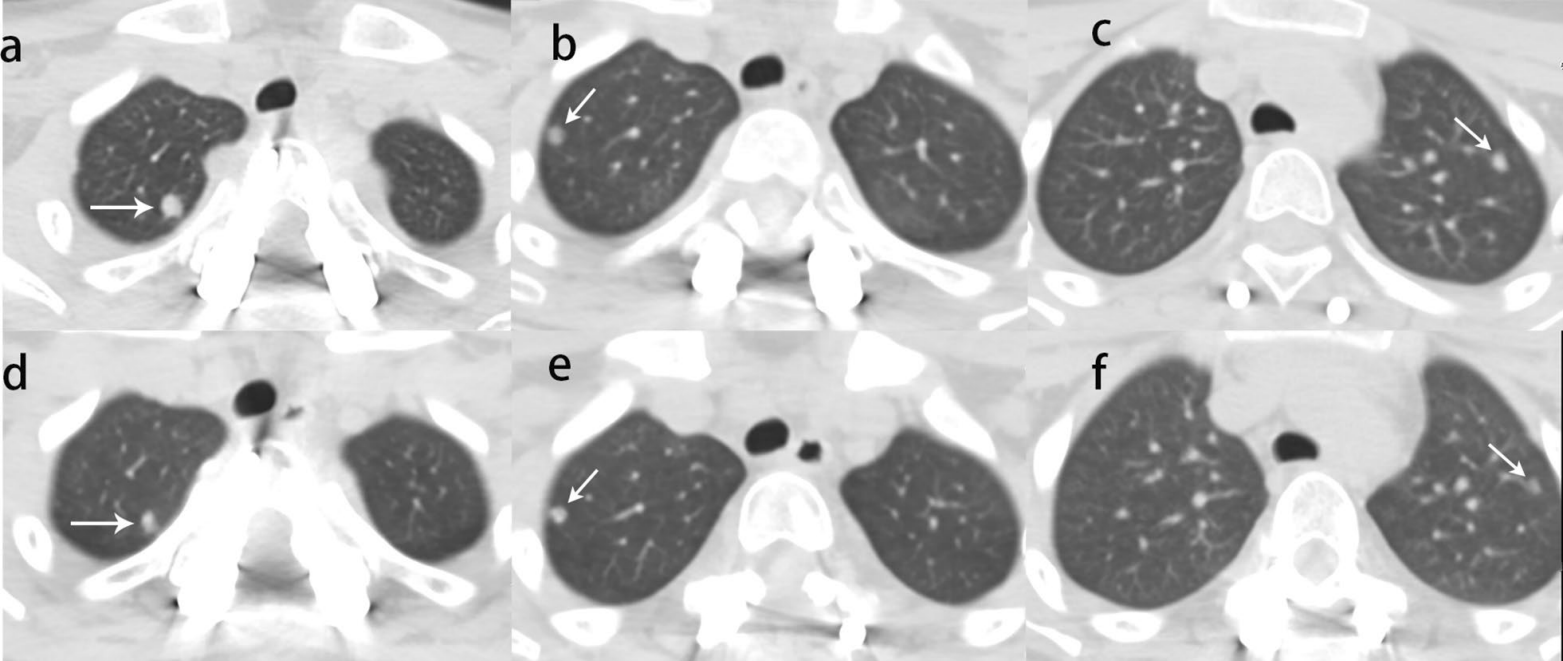
Image of giant cell tumor of T2 in a 13-year-old woman. **a**, **b**, **c** Pre-treatment CT scans showed three lung metastases and the largest diameters was 6.17 mm, 7.19 mm, and 7.92 mm (white arrow). **d**, **e**, **f** After denosumab treatment, lung metastases shrank to 5.09 mm, 6.32 mm, and 5.58 mm, respectively (white arrow)

One patient had three lung metastases with pre-treatment diameters of 6.17 mm, 7.19 mm, and 7.92 mm reduced to 5.09 mm, 6.32 mm, and 5.58 mm after treatment, respectively (Fig. 7). On MR images, a cystic component of the lesion was identified in nine cases, and the mean diameter of the cyst tended to decrease from 13.1 to 12.0 mm, but without a significant difference (Fig. 6). As a measure of vertebral stability, the mean vertebral height was 21.3 mm pretreatment and 20.2 mm after treatment (*p* = 0.007) (Fig. 3b, d). Table 1 shows the radiologic changes after denosumab treatment.

**Table 1:** Radiologic results before and after denosumab treatment

Characteristics	Before treatment	After treatment	*t* or *Z* value	*p* value
Maximum diameter (mm)^†^	52.5 (30.5, 147.0)	48.2 (30.3, 90.1)	− 4.015	< 0.001
Proportion of the spinal canal (%) *	36.8 ± 23.8	18.5 ± 11.9	6.738	< 0.001
Signal intensity (SI) ratio*	0.77 ± 0.22	0.58 ± 0.22	3.754	0.001
Maximum diameter of lung metastases (mm)	6.17, 7.19 and 7.92	5.09, 6.32 and 5.58		
Vertebral height (mm) *	21.3 ± 1.3	20.2 ± 1.4	6.738	0.007
Diameter of the cyst (mm) *	13.1 ± 6.4	12.0 ± 9.6	0.458	0.658
Spinal cord compression	6	3		

^†^The values are given as the median, with the range in parentheses

^*^The values are given as the mean and standard deviation

### Clinical changes

According to the VAS, local pain was relieved in 22 patients (91.7%), while the score for the remaining two patients did not show a significant change. The mean of the VAS scores decreased from pre-treatment 5.3 (range 0–9) to post-treatment 2.0 (range 0–5) (*p* < 0.001). The KPS score increased from a median of 65 (range 40–100) to 80 (range 60–100) (*p* < 0.001). Individually, 17 patients (70.8%) had an elevated score after treatment, and the remaining seven patients had the same score before and after treatment. The ECOG score of eight patients improved (*p* = 0.003). In the six patients with myelopathy, the neurological function of four patients improved according to the Frankel grade (*p* = 0.046) (Table 2).

**Table 2:** Clinical results before and after denosumab treatment

Scoring system	Before treatment	After treatment	*t* or *Z* Value	*p* value
VAS*	5.3 ± 2.7	2.0 ± 1.6	7.032	< 0.001
KPS^†^	65 (40, 100)	80 (60, 100)	− 3.652	< 0.001
ECOG			− 2.972	0.003
0	1	2		
1	12	18		
2	7	4		
3	4	0		
Frankel grade			− 2.000	0.046
D	6	2		
E	18	22		

VAS, Visual Analog Scale; KPS, Karnofsky Performance Scale;ECOG, Eastern Cooperative Oncology Group

^*^The values are given as the mean and standard deviation

^†^The values are given as the median, with the range in parentheses

### Side effects

During denosumab injection, two patients developed mild hypocalcemia at 6 months and 14 months, and one patient developed mild hypophosphatemia at 6 months. No patient was found to have ONJ or other serious adverse effects.

## Discussion

This study adds to the anecdotal evidence regarding denosumab for treating GCTs of the mobile spine by systematically evaluating the radiologic and clinical changes associated with its use in 24 patients from a single institution. The results showed peripheral and internal tumor ossification, tumor shrinkage, intraspinal lesion shrinkage, lung metastasis reduction, and relief of spinal cord compression, all without serious side-effects, after denosumab treatment. There are few studies on the therapeutic effect of denosumab in the previous literature, and most of the cases are giant cell tumors of the limbs. To our knowledge, this is the largest quantitative analysis of the effects of denosumab on GCT of the spine.

It is known that denosumab can promote bone formation. Boriani et al. [14] described bone formation in nine patients with GCTs of the spine after receiving denosumab treatment, with eight patients showing marginal sclerosis and five patients showing internal ossification inside the mass. In the present study, the degree of ossification in 12 patients (50%) was grade 2b and above according to the Boriani grading system. The surgeon also reported a firmer consistency of the tumor capsule during the operation, which facilitated the removal. The mechanism of ossification may be due to a decrease in RANK-positive stromal cells and osteoclast-like giant cells, and the replacement of the tumor with intermixed bone and fibroblast-like spindle cells [19].

The present study adds to the evidence that denosumab can shrink tumors, reduce the occupied area in the spinal canal, and shrink lung metastases. In a previous multi-center retrospective study by Goldschlager et al. [16], the tumor volume in five cases of spinal GCTs decreased by > 10%, and the epidural GCT had a greater regression after 6 months of treatment with denosumab. In the present study, the maximum diameter of the tumors in 21 patients (87.5%) decreased, with the mean maximum diameter reduction of 10.6% (58.3 to 52.1 mm). The proportion of the area of the spinal canal occupied by the tumors decreased in all patients, with the mean reduction of 49.7% (from 36.8 to 18.5%). In addition, the lung metastases in one patient shrank significantly following denosumab treatment, which is consistent with the previous literature [20, 21].

In this study, we observed a significant decrease in the signal intensity ratio between the solid part of the GCTB and the normal spinal cord after denosumab treatment, this may be due to the extensive ossification within the tumor and the reduction in cystic components. Therefore, this radiologic change might be relevant to therapeutic effect. The mean diameter of the cysts was decreased by 8.4% (from 13.1 to 12.0 mm), but the difference was not significant. The mean vertebral height was also reduced, which represented the insufficient stability at the initial stage of treatment.

It has been demonstrated that denosumab can effectively relieve pain and improve the neurological function of patients with spinal GCTs. A phase II clinical study conducted by Martin-Broto et al. [22] showed that most patients with GCTs of the limbs who received denosumab experienced clinically relevant decreases in pain within 2 months. Dubory et al. [18] described the effects of denosumab treatment for GCTs of the spine in eight patients and found that pain and neurologic deficit improved for all patients. In this study, up to 91.7% of patients experienced pain relief after denosumab treatment, 66.7% of patients had improved neurological function according to the Frankel grade, 70.8% of patients had improved KPS scores, and 33.3% of patients improved performance status according to the ECOG score, all with significant differences between pre- and post-treatment measures. These effects may be due to denosumab promoting the healing of bone destruction, reducing the size of the tumor, and relieving the tumor’s compression on the spinal cord and nerve roots. Spinal cord compression disappeared completely in 50% of patients after denosumab treatment in the present study, which is consistent with the improvement of the patients' neurological function.

Side effects associated with denosumab including anemia, nausea, fatigue, muscle and joint pain, pain in the extremities, hypocalcemia, hypophosphatemia, and ONJ have been reported [12, 23]. In this study, 8.3% and 4.2% of patients experienced hypocalcemia and hypophosphatemia, respectively. However, no serious adverse effects were observed, such as ONJ, sarcomatous transformation. This shows the relative safety of denosumab injection for spinal GCTs.

This study had several limitations. First, this was a retrospective analysis from a single-center, which introduced the possibility of selection bias. Second, there were only 24 cases, which is a small sample for robust statistical analysis. Thus, more cases are needed to strengthen the results and the generalizability of the findings. Finally, further pathological studies are recommended to analyze the causes of clinical and radiologic changes.

## Conclusions

Denosumab can be used for GCTs of the mobile spine to relieve pain, improve neurological function, and improve the quality of life. On images, peripheral and internal tumor ossification, tumor shrinkage, intraspinal lesion shrinkage, lung metastasis reduction, spinal cord compression relief and the decrease of SI ratio on T2-weighted image were observed following denosumab treatment. No serious side effects were observed during the application.

## Data Availability

The datasets used and/or analyzed during the current study are available from the corresponding author on reasonable request.

## Abbreviations

CT: Computed tomography
ECOG: Eastern Cooperative Oncology Group
GCT: Giant cell tumor
GCTB: Giant cell tumor of bone
KPS: Karnofsky Performance Scale
MRI: Magnetic resonance imaging
ONJ: Osteonecrosis of the jaw
RANK: Receptor activator of nuclear factor-kappa B
RANKL: Receptor activator of nuclear factor-kappa B ligand
VAS: Visual Analogue Scale
WBB: Weinstein–Boriani–Biagini
